# The Prevalence of Multiple Sclerosis in 3 US Communities: The Role of Vitamin D [Response to Letter]

**Published:** 2010-06-15

**Authors:** Curtis W. Noonan, Dhelia M. Williamson, Judy P. Henry, Laurie Wagner, Ruth Ann Marrie

**Affiliations:** University of Montana, Missoula, Montana; Centers for Disease Control and Prevention, Atlanta, Georgia; Texas Department of Health, Austin, Texas; Texas Department of Health, Austin, Texas; Cleveland Clinic, Cleveland, Ohio

## In Reply:

We appreciate Dr Grant's comments (1) on our descriptive analysis of multiple sclerosis (MS) prevalence in 3 US communities (2). Respectfully, we disagree that we misinterpreted the findings. As researchers, we are cautious about speculating beyond the presented data or offering policy recommendations that are not sufficiently supported by the findings.

Dr Grant focused his comments on 2 aspects of our manuscript. First, he reiterates the similarities between our findings and those by others (3) but criticizes the absence of a detailed comparison between the 2 data sets. The figure presented by Dr Grant supports our findings of a geographic gradient but does little to extend the argument that UV exposure is protective for MS. Dr Grant's argument is based on replacing 1 exposure variable that is assigned at the population level, latitude, with another correlated variable, UV exposure.

Second, Dr Grant notes that we failed to suggest a mechanism for the link. As indicated in our discussion (2), the precise mechanism whereby UV exposure is inversely associated with MS is unknown, but we agree that mounting evidence suggests that vitamin D synthesis may play a role in this pathway (4). Dr Grant further suggests a seasonal interactive effect between UV-dependent vitamin D exposure and the Epstein-Barr virus. We are unclear as to how our manuscript provides a platform for such an argument in the absence of any individual-level exposure data regarding either factor. Dr Grant further argues that the suspected role of vitamin D as a protective factor for the risk of MS should inform the upcoming Institute of Medicine dietary reference intakes for vitamin D. We do not disagree with this assertion, but we trust the Institute of Medicine will consider all available data, including the risks and benefits of changing vitamin D recommendations, in its reassessment of these dietary guidelines.
